# Draft Genome Sequence of Invasive Salmonella enterica Serovar Cannstatt Harboring *mcr-1*.*1*, Isolated from a Fatal Sepsis Case

**DOI:** 10.1128/MRA.01270-20

**Published:** 2021-02-25

**Authors:** Wantana Paveenkittiporn, Watcharaporn Kamjumphol, Anusak Kerdsin

**Affiliations:** a National Institute of Health, Department of Medical Sciences, Ministry of Public Health, Nonthaburi, Thailand; b Faculty of Public Health, Kasetsart University Chalermphrakiat Sakon Nakhon Province Campus, Sakon Nakhon, Thailand; University of Maryland School of Medicine

## Abstract

Here, we report the whole-genome sequence of multidrug-resistant Salmonella enterica serovar Cannstatt harboring *mcr-1.1*, isolated from a fatal sepsis case. Genomic analysis revealed that the isolate was sequence type 2390 carrying *mcr-1.1*, *bla*_CTX-M-14_, *aac(3)IId*, *aac(6′)Iaa*, *floR*, *qnrS1*, *sul2*, *tetA*, and *tetM*. Three Inc plasmids were observed, including the IncX4 plasmid containing *mcr-1.1*.

## ANNOUNCEMENT

Salmonella enterica is a pathogen of substantial concern to global human and animal health (1). Furthermore, multidrug-resistant *Salmonella* bacteria—important agents in the transmission of antibiotic resistance genes—have become a severe problem in the animal breeding sector and a threat to human health (2). In particular, the plasmid-borne genes *mcr-1* to *mcr-10*, which confer resistance to colistin, have been a public health issue since colistin was first administered as a last-resort treatment for Gram-negative bacterial infections (3).

We determined the whole-genome sequence of the invasive S. enterica serovar Cannstatt (isolate no. 130-20), carrying the *mcr-1* gene, isolated from a fatal sepsis case in Thailand. It was isolated from the blood of a 40-year-old man admitted to a tertiary hospital in northern Thailand in October 2019. The isolate was cultured on sheep blood agar at 37°C for 18 hours. The species was identified by conventional biochemical tests and key substrates, such as triple sugar iron agar, lysine indole motility medium, ornithine, urea, and citrate (4). The *Salmonella* serovar was identified by slide agglutination based on the White-Kauffmann-Le Minor scheme (5).

The bacterium was grown on tryptic soy agar at 37°C for 18 hours. Genomic DNA was extracted using ZymoBIOMICS DNA kits (Zymo Research, USA) and quantified using the Invitrogen Qubit double-stranded DNA (dsDNA) high-sensitivity (HS) assay kit (Thermo Fisher Scientific, MA, USA), according to the manufacturers’ protocols. Genomic libraries were generated with the QIAseq FX DNA library kit (Qiagen) following the manufacturer’s protocol. Whole-genome sequencing was performed using the MiSeq platform (Illumina, CA, USA) to obtain 250-bp paired-end reads according to the manufacturer’s instructions. We performed a quality check of the Illumina reads using FastQC (http://www.bioinformatics.babraham.ac.uk/projects/fastqc/) and *de novo* assembled the genome using CLC Genomics Workbench v12.0.2 (CLC Bio, Denmark). Genomic sequences were submitted to the NCBI Prokaryotic Genome Annotation Pipeline (PGAP; v4.12) for annotation. Default parameters were used for all software unless otherwise specified.

This study was reviewed and approved by the Ethics Review Board (ERB) of the Ministry of Public Health, Thailand. The ERB waived the requirement for informed consent because the study satisfied the conditions of the policy statement on ethical conduct for research involving humans. This study was conducted according to the principles of the Declaration of Helsinki.

A total of 2,065,260 raw reads were obtained for S. enterica serovar Cannstatt 130-20. After *de novo* reconstruction, 69 contigs were assembled with an *N*_50_ value of 176,525 bp. On average, the assembled draft genome was covered 52.07 times. The draft genome size was determined to be 5,069,457 bp, and the GC content was 51.8%. Genomic identification of S. enterica was evidenced using both average nucleotide identity (ANI) (6) analysis and Kraken2 v2.0.9 (7). Analysis of the serovar using *Salmonella In Silico* Typing Resource (SISTR) identified it as *S. enterica* serovar Cannstatt (8). It was sequence type 2390 (ST2390) and core genome sequence type (cgST) 194255 according to MLST 2.0 (9) and cgMLSTFinder 1.1, respectively (10). The isolate genome included acquired antimicrobial resistance genes, namely, *mcr-1.1*, *bla*_CTX-M-14_, *aac(3)IId*, *aac(6′)Iaa*, *floR*, *qnrS1*, *sul2*, t*etA*, and *tetM*, according to ResFinder (11) and CARD (12). A mutation in *parC* at position T57S was observed, indicating that the isolate may be nonsusceptible to nalidixic acid and ciprofloxacin, while the *parE*, *gyrB*, and *gyrA* genes showed no mutations (11). The PlasmidFinder tool revealed three Inc group plasmids, namely, IncFIA(HI1), IncHI1A, and IncX4 (13), and *mcr-1.1* was observed in the IncX4 plasmid.

### Data availability.

The results of this whole-genome shotgun project were deposited in DDBJ/ENA/GenBank under the BioProject no. PRJNA525849, BioSample no. SAMN14997812, and accession no. JABMLN000000000. The Sequence Read Archive (SRA) number is SRR12897514.
